# Regulatory T Cells and Tumor-Associated Macrophages in the Tumor Microenvironment in Non-Muscle Invasive Bladder Cancer Treated with Intravesical Bacille Calmette-Guérin: A Long-Term Follow-Up Study of a Japanese Cohort

**DOI:** 10.3390/ijms18102186

**Published:** 2017-10-19

**Authors:** Makito Miyake, Yoshihiro Tatsumi, Daisuke Gotoh, Sayuri Ohnishi, Takuya Owari, Kota Iida, Kenta Ohnishi, Shunta Hori, Yosuke Morizawa, Yoshitaka Itami, Yasushi Nakai, Takeshi Inoue, Satoshi Anai, Kazumasa Torimoto, Katsuya Aoki, Keiji Shimada, Noboru Konishi, Nobumichi Tanaka, Kiyohide Fujimoto

**Affiliations:** 1 Department of Urology, Nara Medical University, 840 Shijo-cho, Kashihara-shi, Nara 634-8522, Japan; takuro.birds.nest@gmail.com (Y.T.); dgotou@gmail.com (D.G.); sayuri3@naramed-u.ac.jp (S.O.); tintherye@gmail.com (T.O.); kota1006ida@yahoo.co.jp (K.I.); kenzmedico0912@yahoo.co.jp (K.O.); horimaus@gmail.com (S.H.); tigers.yosuke@gmail.com (Y.M.); y.itami.324@gmail.com (Y.I.); nakaiyasusiuro@live.jp (Y.N.); you1513tt@yahoo.co.jp (T.I.); sanai@naramed-u.ac.jp (S.A.); torimoto@nmu-gw.naramed-u.ac.jp (K.T.); aokik@naramed-u.ac.jp (K.A.); sendo@naramed-u.ac.jp (N.T.); kiyokun@naramed-u.ac.jp (K.F.); 2 Department of Pathology, Nara Medical University, 840 Shijo-cho, Kashihara-shi, Nara 634-8522, Japan; n-konishi@takai-hp.com; 3 Department of Pathology, Nara City Hospital, 1501 Higashi kidera-cho, Nara-shi, Nara 630-8305, Japan; k-shimada@nara-jadecom.jp

**Keywords:** regulatory T cell, tumor-associated macrophage, non-muscle invasive bladder cancer, intravesical recurrence, progression, Bacille Calmette–Guérin

## Abstract

The clinical significance of regulatory T cells (Treg) and tumor-associated macrophages (TAM) in the tumor microenvironment of human bladder cancer remains unclear. The aim of this study is to explore their relevance to oncological features in non-muscle invasive bladder cancer (NMIBC). We carried out immunohistochemical analysis of forkhead box P3 (FOXP3, Treg maker), CD204 (TAM marker), and interleukin-6 (IL6) using surgical specimens obtained from 154 NMIBC patients. The Treg and TAM counts surrounding the cancer lesion and IL6-positive cancer cell counts were evaluated against clinicopathological variables. We focused on the ability of the Treg and TAM counts around the cancer lesion to predict outcomes after adjuvant intravesical Bacille Calmette–Guérin (BCG) treatment. High Treg counts were associated with female patients, older age, T1 category, and high tumor grade. TAM count was significantly correlated with Treg count and with IL6-positive cancer cell count. In our analysis of 71 patients treated with BCG, high counts of Treg and TAM were associated with shorter recurrence-free survival, and the former was an independent predictor of recurrence. Poor response to intravesical BCG was associated with Treg and TAM in the tumor microenvironment. Disrupting the immune network can be a supplementary therapeutic approach for NMIBC patients receiving intravesical BCG.

## 1. Introduction

Urothelial carcinoma (UC) of the bladder is a heterogeneous disease in terms of its clinical and biological aspects [1]. Among non-muscle invasive bladder cancer (NMIBC) patients, several factors such as age, T category, tumor grade, tumor size, and multiplicity are recognized to predict the risk of intravesical recurrence and progression after transurethral resection of bladder tumor (TURBT) [2,3]. Despite advancements in detection technologies, surgical techniques, and adjuvant intravesical treatment, clinical management of high-risk diseases remains challenging [4,5].

Intravesical administration of Bacille Calmette–Guérin (BCG) is a standard treatment for carcinoma in situ (CIS) and an adjuvant option for T1 and high-risk Ta tumors following TURBT [6]. However, a delay in radical cystectomy (RC) leads to shortened cancer-specific survival as compared to immediate RC at the time of NMIBC [7]. Thus, there is an urgent need for biomarkers to identify patients who will benefit from intravesical BCG or should undergo immediate RC. Although possible mechanisms underlying BCG-induced antitumor activity and relevant molecules have been studied [8,9,10], they are not fully understood. Briefly, BCG is internalized into urothelial cells through the formation of a complex with fibronectin, followed by antigen presentation to BCG-specific CD4+ T-cells by antigen-presenting cells. Pro-inflammatory cytokines such as interleukin-6 (IL6) and interferon-γ are secreted to recruit a Th1-induced immunoreaction with the recognition of cancer cells through the activation of macrophages, CD8+ T-cells, and natural killer cells.

Several previous studies have demonstrated that the pre-BCG baseline status of Th1/Th2 balance, regulatory T cell (Treg) recruitment, and tumor-associated macrophage (TAM) polarization in the tumor microenvironment could influence the clinical response to BCG [10,11,12]. In light of the sparse data on the clinical significance of Treg/TAM and their relevance to oncological outcomes of NMIBC treated with intravesical BCG—especially in Japanese patients—this study was conducted with the goal of improving patient care.

## 2. Results

### 2.1. Association of Treg and TAM in the Cancerous Area with Baseline Characteristics

**Figure 1:**
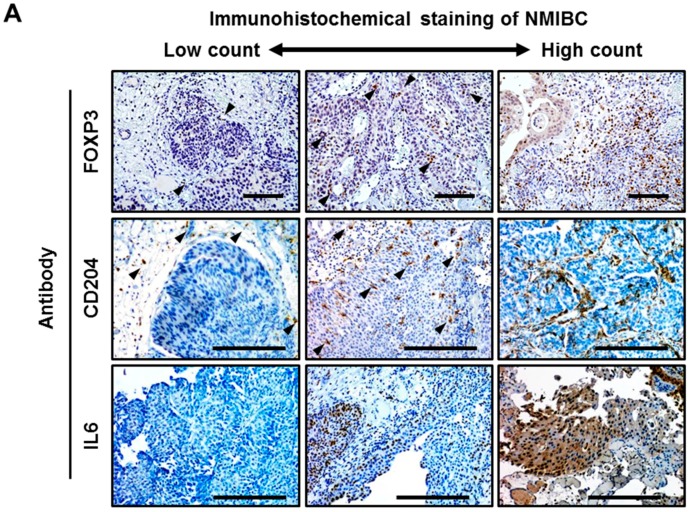

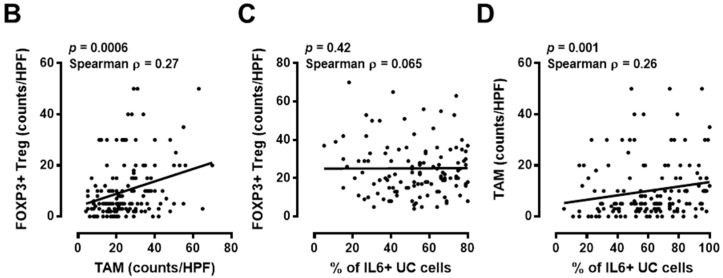
Immunohistochemical quantification of regulatory T cells, tumor-associated macrophages, and IL6-positive urothelial carcinoma cells. (**A**) Representative expression status of FOXP3, CD204, and IL6 in human bladder cancer tissues. Images were captured at 100× (FOXP3) or 200× (CD204 and IL6) magnification. Black arrowheads in the FOXP3 and CD204 images indicate positive-stained immune cells that exist in the stroma near the cancer cells or filtrate to the tumors. Scale bars, 200 μm. The interrelationship between (**B**) the Treg counts and TAM counts, (**C**) the Treg counts and the percentage of IL6^+^ cancer cells, and (**D**) the TAM counts and the percentage of IL6+ cancer cells were examined using Spearman’s correlation. HPF, high power field; Treg, regulatory T cell; TAM, tumor-associated macrophage; UC, urothelial carcinoma.

Treg and TAM significantly differed in their localization patterns (Figure 1A). Most of the Treg cells localized in the stroma around the cancer lesion regardless of tumor stage and grade, whereas TAM had a tendency to infiltrate into the tumor area in high-grade tumors compared to low-grade tumors. In an analysis of 154 tumors, the median counts per high power field (HPF) of Treg and TAM were eight (interquartile range [IQR], 3–15) and 23 (IQR 16–33), respectively. The appropriate cutoff points of Treg and TAM count have not been established yet, especially in bladder UC. The cutoff values for separating low counts and high counts for Treg and TAM were set as 10 and 25 respectively, based on the median values. With these thresholds, high counts of Treg and TAM were found in 68 (44%) and 62 (41%) out of 154 tumors, respectively. The clinicopathological variables and their association with Treg and TAM in 154 patients with NMIBC are outlined in Table 1. High Treg count was associated with female patients (*p* = 0.001), older age (*p* = 0.024), T1 category (*p* < 0.001), high tumor grade (*p* < 0.001), and the presence of CIS (*p* = 0.011), whereas the count of TAM was not associated with any variable (Table 1).

### 2.2. Correlation among Treg, TAM, and IL6 in the Bladder Tumor Microenvironment

IL6 is one of the major pro-inflammatory cytokines in the tumor microenvironment, and promotes cancer progression and therapeutic resistance [13]. To investigate the correlation between Treg, TAM, and IL6+ UC cells, the Spearman correlation coefficient was analyzed among the three markers. There was a weak positive correlation between the counts of Treg and TAM (*p* < 0.001, Figure 1B). The count of TAM, but not Treg (*p* = 0.42, Figure 1C), positively correlated with counts of IL6+ cancer cells (*p* = 0.001, Figure 1D).

### 2.3. Prognostic Role of Baseline Treg and TAM in NMIBC Treated with Intravesical BCG

**Figure 2:**
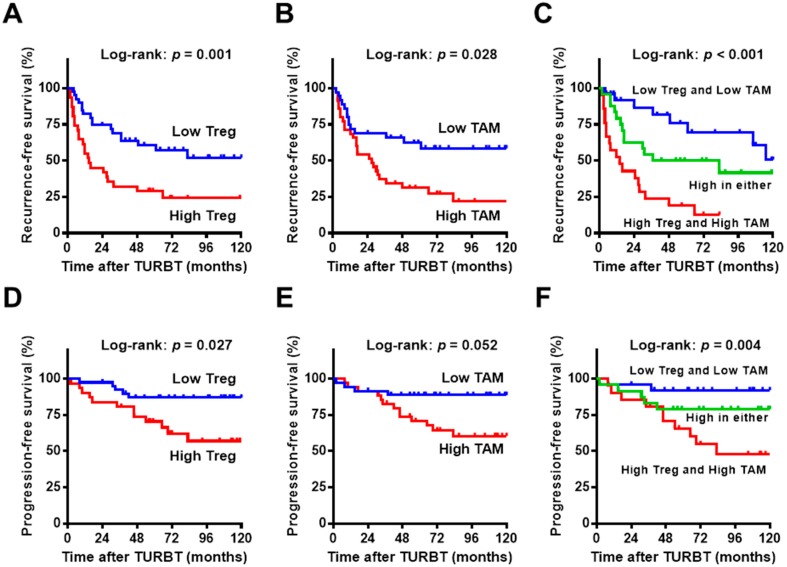
Kaplan–Meier plots for 71 patients treated with intravesical BCG. Intravesical recurrence-free survival (**A**–**C**) and progression-free survival (**D**–**F**) after initial TURBT are plotted. (**A**,**D**) Survival curves according to the Treg count, low (<10 cells/HPF) vs. high (≥10 cells/HPF); (**B**,**E**) survival curves according to the TAM count, low (<25 cells/HPF) vs. high (≥25 cells/HPF); (**C**,**F**) survival curves according to the number of immune cells with high counts (0, blue; 1, green; 2, red). The log-rank test was used for comparison. Treg, regulatory T cell; TAM, tumor-associated macrophage. Author 1, A.B. Title of Thesis. Level of Thesis, Degree-Granting University, Location of University, Date of Completion.

In the analysis of 71 patients treated with intravesical BCG, 40 (56%) had recurrence and 17 (24%) developed progression after a median follow-up period of 83 (IQR 61–115) months. To examine the relevance of the studied variables and prognosis, we performed univariate and multivariate analyses for recurrence-free survival (RFS) and progression-free survival (PFS) (Table 2). High counts of Treg and TAM and low tumor grade were associated with shorter recurrence-free survival in the univariate analysis (Figure 2A,B,D,E), while high count of Treg (*p* = 0.001, hazard ratio (HR) = 3.07, vs. low count) and low tumor grade (*p* = 0.01, HR = 0.27, vs. high tumor grade) were identified as independent predictors for recurrence. An additional analysis revealed that a high count of Treg was an independent predictor for progression (*p* = 0.021, HR = 3.43, vs. low count), whereas high counts of TAM showed marginal association with short PFS in the univariate analysis (*p* = 0.052, HR = 3.35, vs. low count). When patients were stratified into three groups according to the counts of Treg and TAM, 26 (37%) patients exhibited low counts of both, 24 (34%) exhibited a high count of either Treg or TAM, and the remaining 21 (29%) exhibited high counts of both. Both RFS and PFS decreased dramatically as the number of high counts of immune cells increased (Figure 2C,F).

## 3. Discussion

This study demonstrates the association between poor responses to intravesical BCG and both Treg and TAM in the tumor microenvironment of human bladder UC. Although it has been decades since intravesical BCG was first introduced for bladder UC treatment in 1976 [14], no molecular biomarkers are available in clinical settings for predicting responses. There is currently little clinical evidence about associations between these tumor-supporting immune cells and resistance to intravesical BCG. A recent report by Pichler et al. investigated the baseline immune environment in NMIBC treated with intravesical BCG by IHC analysis using a total of 10 antibodies. The authors concluded that increased counts of CD4+ and GATA3+ T-cells were associated with prolonged RFS, whereas high counts of TAM and Treg were associated with shortened RFS [10]. Suriano et al. performed detailed population analyses of macrophages in the bladder tumor environment using dual immunofluorescence staining using CD68/iNOS (M1 polarization) and CD68/CD163 (M2 polarization) combinations, suggesting a prognostic value of TAM infiltration for RFS in patients with NMIBC treated with intravesical BCG [12]. One of the major drawbacks in both of these reports is the limited number of patients (only 40 each), which did not allow for multivariate analysis providing reliable prognostic values. Moreover, the topical immune environment, response pattern toward exposure of BCG, and the strains of BCG used can vary among races and countries. Therefore, we conducted the present study to explore the relevance to oncological features in a larger sample size and a Japanese cohort.

Immune cells in the tumor area or in the stroma around the tumor area can influence survival, with either poor or improved prognosis and with either sensitivity or resistance to treatment, depending on their subsets and polarization [10]. Muscle-invasive bladder cancer (MIBC) patients with FOXP3 expression in tumor cells showed shorter survival compared to those with negative cancers [15]. Another report demonstrated that an elevated FOXP3/CD8 ratio in tumor tissues was an independent predictor of poor prognosis after RC [16]. In a multivariate analysis of MIBCs, a high count of CD68+ TAM was correlated with high T category, high-grade cancer [17], and higher risk of cancer-specific death when adjusted for CD3 [18]. Although extensive studies on muscle-invasive bladder cancer have been conducted, there is little information on NMIBC, especially about its response to intravesical BCG. The present study demonstrated that high counts of Treg and TAM acted as a predictor of poor prognosis with a 2.5-fold and 2.3-fold higher risk of recurrence and a 3.38-fold and 3.35-fold higher risk of progression in univariate analysis, respectively (Table 2). These results strongly suggest a possible relationship between resistance to BCG and Treg/TAM in the tumor microenvironment.

Treg plays crucial roles in the evasion of antitumor immunity and escape from response to treatments in various malignancies, leading to poor oncological outcomes [10,19,20]. BCG treatment is known to cause Th1-polarized immunomodulation [21]. Treg can suppress effector mechanisms of the immune response in vaccination models. However, simultaneous inhibition of Th2 polarization and Tregs could promote host-protective immunity [22] and BCG-induced vaccination response using a mouse model through enhanced Th1 polarization [23]. Many studies have demonstrated that TAM cells are markedly present in various malignancies and involved in promoting neoangiogenesis and producing immunoregulatory and immunosuppressive cytokines, culminating in worsened oncological outcomes [24]. Previous studies have stated that the predominant locations of TAM are the stroma and lamina propria [10,25]. However, our analysis showed that a large number of TAM localize in the tumor area in high-grade tumors compared to low-grade tumors. Ayari et al. demonstrated that CD68+ TAM in the stroma or within tumor nests were found to have no predictive value for outcomes after intravesical BCG [26]. Further studies with larger sample sizes are needed to clarify the influence of the predominant location of Treg and TAM on prognostic significance.

IL6 is one of the major pro-inflammatory cytokines in the tumor microenvironment, and exhibits tumor-supporting activities [13,27,28]. In the present study, we examined the correlation between Treg/TAM and IL6 in the bladder tumor microenvironment. There was a positive correlation between the count of TAM and IL6+ UC cells, whereas significant correlation was not observed between the counts of Treg and IL6+ UC cells (Figure 1C,D). This finding is consistent with evidence from previous studies. Hasita et al. investigated the significance of TAM and Treg in patients with intrahepatic cholangiocarcinoma, demonstrating that IL6 production from tumor cells was correlated with the number of infiltrating TAMs, but not with the numbers of Treg cells or vessels [27]. This result supports the idea that IL6 is one of the vital molecules that differentiates macrophages. Another report by Hinz et al. revealed that specific down-regulation of FOXP3 with small interfering RNA in the pancreatic carcinoma cell line Panc89 resulted in the up-regulation of IL6 and IL8 via the activation of nuclear factor-κB (NF-κB), providing evidence for the control of inflammatory cytokine production by FOXP3 [28]. IL8 promotes angiogenesis and growth of cancer cells. However, the biological significance of FOXP3-mediated suppression of IL6 and IL8 production in malignant diseases is not fully understood.

The present study has several limitations. The first is its retrospective nature with potential selection bias; for example, some patients were excluded because of aggressive treatment (immediate radical cystectomy for T1 disease). Second, tumor tissue analysis was performed by immunohistochemistry (IHC) with possible technical biases, for example specimen fixation, antigen retrieval, antibody binding, color development, and quantification, which may affect the interpretation. Third, this study includes 71 patients, which is considered to be a relatively low sample size. Because low sample size constitutes a limitation of the work and to acknowledge this issue, further study including additional tumors and patients is needed to verify our results.

## 4. Materials and Methods

### 4.1. Data Collection of the Patients

All subjects gave their informed consent for inclusion before they participated in the study. The study was conducted in accordance with the Declaration of Helsinki, and the protocol was approved by the Ethics Committee of the Nara Medical University (Project identification code: 1630, accepted: 21 August 2017). In total, 154 patients with newly diagnosed NMIBC undergoing TURBT between 2004 and 2013 were enrolled in this study. Clinical information was retrieved from medical charts. All hematoxylin and eosin-stained specimens obtained by initial TURBT were reassessed independently by two experienced uropathologists (Keiji Shimada and Noboru Konishi) to determine T category (2010 American Joint Committee on Cancer TNM Staging system), tumor grade (2004 WHO classification), CIS, and lymphovascular invasion. Follow-up was performed according to our institutional protocol [29].

### 4.2. Immunohistochemical Staining and Quantification

IHC staining of paraffin-embedded, formalin-fixed tissue blocks was performed using the Histofine SAB-PO kit (Nichirei Co., Tokyo, Japan) as previously described [30,31]. Briefly, the sections were autoclaved for 10 min in 0.01 M citrate buffer (pH 6.0) for antigen retrieval. The primary antibodies were monoclonal mouse anti-FOXP3 (dilution 1/100; Ref. ab20034, Abcam, Cambridge, MA, USA), monoclonal mouse anti-MRS-A (CD204) (dilution 1/2000; Ref. KT022; Trans Genic Inc., Kobe, Japan), and polyclonal rabbit anti-IL6 (dilution 1/500; Ref. sc-1265, Santa Cruz, Dallas, TX, USA).

FOXP3-positive Treg was quantified as previously described [32]. Lymphocytes exhibiting nuclear immunostaining for FOXP3 in the cancerous area were counted in at least five independent HPFs (400×, 0.0625 μm^2^). The mean count of each patient was determined by dividing the sum by the number of assessed fields. Similarly, CD204^−^positive TAM in the cancerous area was counted [30]. To quantify the expression level of IL6 in the UC cells, immunoreactive tumor cells were counted in at least five independent fields, and the percentage of positive cells was calculated by dividing that number by the total counted UC cells (1–100%) [30]. The median values (IQR) of total count cells for the quantification of the FOXP3-positive Treg, CD204-positive TAM, and IL6+ cells were 185 (132–125), 185 (132–125), and 221 (158–291), respectively. Evaluation was carried out by two trained investigators (M.M. and Y.T.) in a blind manner, without knowledge of the patients’ outcome or other clinicopathological characteristics.

### 4.3. Adjuvant Intravesical Therapy for NMIBC after TURBT

TURBT was carried out according to a standardized procedure used by all surgeons at the single institute [29]. Because of the study’s retrospective nature, the criteria, dosage, and scheme for adjuvant intravesical therapy were not consistent between patients, and depended on the physician’s decision. In general, patients with high-risk NMIBC, such as those positive for concomitant CIS, T1 category, and high-grade tumors, were treated with intravesical BCG. A substantial number of patients were given intravesical BCG as initial adjuvant therapy. The schedule for intravesical BCG consisted of weekly instillations for 6–8 consecutive weeks of Immunobladder (BCG Tokyo 172 strain; Japan BCG Laboratory Tokyo, Japan) or ImmuCyst (Connaught strain; Sanofi, Paris, France; currently not being supplied as of October 2017). A single immediate post-TURBT chemotherapy instillation and/or maintenance chemotherapy instillation using anthracyclines or mitomycin-C was given to a subset of the cohort.

### 4.4. Statistical Analysis

The clinicopathological characteristics were compared using the Mann–Whitney *U* test and Fisher’s exact test as appropriate. The interrelationships among the studied parameters were examined using the Spearman correlation coefficient and linear regression analysis. RFS and progression-free survival were calculated from the date of TURBT for the initial TURBT to the date of intravesical recurrence and progression, respectively. Progression was defined as recurrent disease when there was invasion into the muscularis propria (≥T2), lymph node involvement, and/or occurrence of distant metastases. Survival rates were analyzed using the Kaplan–Meier method and compared using the log-rank test for univariate analysis. Multivariate analysis was used to identify independent prognostic variables using a stepwise Cox proportional hazards regression model. IBM SPSS version 21 (SPSS, Inc., Chicago, IL, USA) and PRISM software version 7.00 (GraphPad Software, Inc., San Diego, CA, USA) were used for statistical analyses and data plotting, respectively. Statistical significance in this study was set at *p* < 0.05, and all reported *p* values were two-sided.

## 5. Conclusions

We explored the clinical significance of Treg and TAM in the bladder tumor environment, suggesting that the immunological response to intravesical BCG is a complicated mechanism involving multiple subpopulations of immune cells. Moreover, IL6 production from UC cells might play a key role in the induction of TAM and the protection of the cancer cells from BCG treatment. Therefore, disrupting the recruitment of Treg and TAM, in combination with conventional intravesical BCG could be a potential therapeutic approach. Randomized control trials are required to determine the true clinical benefit.

## Figures and Tables

**Table 1:** Clinicopathologic variables and association with Treg and TAM in primary NMIBC.

Variables	N	Treg (FOXP3+ Cell)			TAM (CD204+ Cell)		
		Low	High	*p* Value	Low	High	*p* Value
Total	154 (100%)	86 (56%)	68 (44%)	−	92 (59%)	62 (41%)	−
Sex				0.0012			0.45
Male	137 (89%)	83 (61%)	54 (39%)		78 (57%)	59 (43%)	
Female	17 (11%)	3 (18%)	14 (82%)		8 (47%)	9 (53%)	
Age at initial TURBT							
categorical				0.056			0.75
<60	18 (12%)	15 (83%)	3 (17%)		11 (61%)	7 (39%)	
60 to 70	55 (36%)	34 (62%)	21 (38%)		32 (58%)	23 (42%)	
>70	81 (52%)	43 (53%)	38 (47%)		43 (53%)	38 (47%)	
Continuous							
median (IQR)	71 (65−76)	69 (63−76)	73 (69−79)	0.024	71 (64−76)	71 (68−77)	0.22
T category				<0.001			0.25
Ta	68 (44%)	52 (76%)	16 (24%)		41 (60%)	27 (40%)	
T1	73 (47%)	30 (41%)	43 (59%)		36 (49%)	37 (51%)	
Tis	13 (9%)	10 (77%)	3 (23%)		9 (69%)	4 (31%)	
Tumor grade				<0.001			0.16
Low	71 (46%)	53 (75%)	18 (25%)		44 (62%)	27 (38%)	
High	83 (54%)	39 (47%)	44 (53%)		42 (51%)	41 (49%)	
Tumor architecture				0.98			0.69
Papillary	134 (87%)	80 (60%)	54 (40%)		74 (55%)	60 (45%)	
Non-papillary	20 (13%)	12 (60%)	8 (40%)		12 (60%)	8 (40%)	
Multiplicity				0.64			0.71
Single	88 (57%)	54 (61%)	34 (39%)		48 (55%)	40 (45%)	
Multiple	66 (43%)	38 (58%)	28 (42%)		38 (58%)	28 (42%)	
Tumor size				0.45			0.99
Less than 3 cm	119 (77%)	73 (61%)	46 (39%)		69 (58%)	50 (42%)	
3 cm or more	35 (23%)	19 (54%)	16 (46%)		17 (49%)	18 (51%)	
CIS				0.011			0.29
No	91 (59%)	62 (68%)	29 (32%)		54 (59%)	37 (41%)	
Yes	63 (41%)	30 (48%)	33 (52%)		32 (51%)	31 (49%)	
LVI (in T1 tumor, *n* = 73)				0.66			0.93
Negative	49 (67%)	21 (43%)	28 (57%)		24 (49%)	25 (51%)	
Positive	24 (33%)	9 (38%)	15 (62%)		12 (50%)	12 (50%)	
Intravesical adjuvant therapy				0.65			0.37
No	64 (42%)	41 (64%)	23 (36%)		40 (62%)	24 (38%)	
BCG	71 (46%)	40 (56%)	31 (44%)		36 (51%)	35 (49%)	
Chemotherapy	19 (12%)	11 (58%)	8 (42%)		10 (53%)	9 (47%)	

Treg, regulatory T cell; TAM, Tumor-associated macrophage; NMIBC, non-muscle invasive bladder cancer; TURBT, transurethral resection of bladder tumor; IQR, interquartile range; CIS, Carcinoma in situ; LVI, lymphovascular invasion; BCG, Bacillus Calmette-Guerin.

**Table 2:** The prognostic factors for recurrence and progression in 71 NMIBC patients treated with BCG.

Variables	N	Intravesical Recurrence-Free Survival						Progression-Free Survival					
		Univariate			Multivariate ^†^			Univariate			Multivariate ^†^		
		HR	95% CI	*p* Value	HR	95% CI	*p* Value	HR	95% CI	*p* Value	HR	95% CI	*p* Value
Sex													
Male	63 (89%)	1						1					
Female	8 (11%)	0.64	0.24–1.72	0.52	NA			1.24	0.25–6.21	0.98	NA		
Age													
≤70	37 (52%)	1						1					
>70	34 (48%)	1.05	0.56–1.97	0.91	NA			1.88	0.73–4.86	0.22	NA		
T stage													
Ta or isolated Tis	30 (42%)	1						1					
T1	41 (58%)	1.20	0.64–2.26	0.69	NA			1.29	0.49–3.40	0.81	NA		
Tumor grade													
Low	13 (18%)	1			1			1					
High	58 (82%)	0.68	0.26–0.96	0.04	0.81	0.24–1.16	0.10	1.85	0.56–6.09	0.48	NA		
Multiplicity													
Single	38 (54%)	1						1					
Multiple	33 (46%)	0.74	0.39–1.39	0.78	NA			1.20	0.46–3.15	0.46	NA		
Tumor size													
<3 cm	52 (73%)	1						1					
≥3 cm	19 (27%)	0.76	0.38–1.52	0.58	NA			1.05	0.39–2.87	0.89	NA		
Concomitant CIS													
No	22 (31%)	1						1					
Yes	49 (69%)	0.48	0.24–1.06	0.097	NA			2.48	0.91–6.74	0.14	NA		
Treg													
Low	31 (44%)	1			1			1			1		
High	40 (56%)	2.53	1.32–4.86	0.001	3.07	1.55–6.07	0.001	3.38	1.29–8.88	0.027	3.43	1.20–9.74	0.021
TAM													
Low	35 (49%)	1			1			1			1		
High	36 (51%)	2.31	1.27–4.30	0.029	1.39	0.68–2.84	0.37	3.35	1.29–8.66	0.052	2.50	0.79–8.02	0.12
IL6+ UC cells													
Low	32 (45%)	1						1					
High	39 (55%)	1.36	0.73–2.56	0.22	NA			1.39	0.53–3.61	0.54	NA		

NMIBC, non-muscle invasive bladder cancer; BCG, Bacillus Calmette-Guerin; HR, hazard ratio; CI, confidence interval; CIS, Carcinoma in situ; Treg, regulatory T cell; TAM, Tumor-associated macrophage; UC, urothelial carcinoma; ^†^ Multivariate Cox regression analysis; NA, not analyzed.

## Abbreviations

BCG	Bacille Calmette–Guérin
CIS	Carcinoma in situ
FR	Hazard ratio
FOXP3	Forkhead box P3
HPF	High-power microscopic fields
IHC	Immunohistochemistry
IL6	Interleukin-6
LVI	Lymphovascular invasion
MIBC	Muscle-invasive bladder cancer
NF-κB	Nuclear factor-κB
NMIBC	Non-muscle invasive bladder cancer
PFS	Progression-free survival
RC	Radical cystectomy
RFS	Recurrence-free survival
TAM	Tumor-associated macrophage
TURBT	Transurethral resection of bladder tumor
Treg	Regulatory T cell
UC	Urothelial carcinoma

## References

[1] Miyake M., Fujimoto K., Hirao Y. (2016). Active surveillance for nonmuscle invasive bladder cancer. Investig. Clin. Urol..

[2] Fernandez-Gomez J., Madero R., Solsona E. (2009). Predicting nonmuscle invasive bladder cancer recurrence and progression in patients treated with bacillus Calmette-Guerin: The CUETO scoring model. J. Urol..

[3] Sylvester R.J., van der Meijden A.P., Oosterlinck W. (2006). Predicting recurrence and progression in individual patients with stage Ta T1 bladder cancer using EORTC risk tables: A combined analysis of 2596 patients from seven EORTC trials. Eur. Urol..

[4] Witjes J.A., Compérat E., Cowan N.C., De Santis M., Gakis G., Lebret T., Ribal M.J., Van der Heijden A.G., Sherif A., European Association of Urology (2014). EAU guidelines on muscle-invasive and metastatic bladder cancer: Summary of the 2013 guidelines. Eur. Urol..

[5] Reis L.O., Moro J.C., Ribeiro L.F., Voris B.R., Sadi M.V. (2016). Are we following the guidelines on non-muscle invasive bladder cancer?. Int. Braz. J. Urol..

[6] Lamm D.L., Blumenstein B.A., Crawford E.D. (1991). A randomized trial of intravesical doxorubicin and immunotherapy with bacilli Calmette-Guerin for transitional-cell carcinoma of the bladder. N. Engl. J. Med..

[7] Raj G.V., Herr H., Serio A.M., Donat S.M., Bochner B.H., Vickers A.J., Dalbagni G. (2007). Treatment paradigm shift may improve survival of patients with high risk superficial bladder cancer. J. Urol..

[8] Kitamura H., Tsukamoto T. (2011). Immunotherapy for urothelial carcinoma: Current status and perspectives. Cancers.

[9] Abebe F. (2012). Is interferon-gamma the right marker for bacilli Calmette-Guérin-induced immune protection? The missing link in our understanding of tuberculosis immunology. Clin. Exp. Immunol..

[10] Pichler R., Fritz J., Zavadil C., Schäfer G., Culig Z., Brunner A. (2016). Tumor-infiltrating immune cell subpopulations influence the oncologic outcome after intravesical Bacillus Calmette-Guérin therapy in bladder cancer. Oncotarget.

[11] Nunez-Nateras R., Castle E.P., Protheroe C.A., Stanton M.L., Ocal T.I., Ferrigni E.N., Ochkur S.I., Jacobsen E.A., Hou Y.X., Andrews P.E. (2014). Predicting response to bacillus Calmette-Guérin (BCG) in patients with carcinoma in situ of the bladder. Urol. Oncol..

[12] Suriano F., Santini D., Perrone G., Amato M., Vincenzi B., Tonini G., Muda A., Boggia S., Buscarini M., Pantano F. (2013). Tumor associated macrophages polarization dictates the efficacy of BCG instillation in non-muscle invasive urothelial bladder cancer. J. Exp. Clin. Cancer Res..

[13] Kumari N., Dwarakanath B.S., Das A., Bhatt A.N. (2016). Role of interleukin-6 in cancer progression and therapeutic resistance. Tumour Biol..

[14] Morales A., Eidinger D., Bruce A.W. (1976). Intracavitary Bacillus Calmette-Guerin in the treatment of superficial bladder tumors. J. Urol..

[15] Winerdal M.E., Marits P., Winerdal M., Hasan M., Rosenblatt R., Tolf A., Selling K., Sherif A., Winqvist O. (2011). FOXP3 and survival in urinary bladder cancer. BJU Int..

[16] Horn T., Laus J., Seitz A.K., Maurer T., Schmid S.C., Wolf P., Haller B., Winkler M., Retz M., Nawroth R. (2016). The prognostic effect of tumour-infiltrating lymphocytic subpopulations in bladder cancer. World J. Urol..

[17] Boström M.M., Irjala H., Mirtti T., Taimen P., Kauko T., Ålgars A., Jalkanen S., Boström P.J. (2015). Tumor-Associated Macrophages Provide Significant Prognostic Information in Urothelial Bladder Cancer. PLoS ONE.

[18] Sjödahl G., Lövgren K., Lauss M., Chebil G., Patschan O., Gudjonsson S., Månsson W., Fernö M., Leandersson K., Lindgren D. (2014). Infiltration of CD3+ and CD68+ cells in bladder cancer is subtype specific and interacts the outcome of patients with muscle-invasive tumors. Urol. Oncol..

[19] Mahmoud S.M., Paish E.C., Powe D.G., Macmillan R.D., Lee A.H., Ellis I.O., Green A.R. (2011). An evaluation of the clinical significance of FOXP3+ infiltrating cells in human breast cancer. Breast Cancer Res. Treat..

[20] Shimizu K., Nakata M., Hirami Y., Yukawa T., Maeda A., Tanemoto K. (2010). Tumor-infiltrating FOXP3+ regulatory T cells are correlated with cyclooxygenase-2 expression and are associated with recurrence in resected non-small cell lung cancer. J. Thorac. Oncol..

[21] Ponticiello A., Perna F., Maione S., Stradolini M., Testa G., Terrazzano G., Ruggiero G., Malerba M., Sanduzzi A. (2004). Analysis of local T lymphocyte subsets upon stimulation with intravesical BCG: A model to study tuberculosis immunity. Respir. Med..

[22] Bhattacharya D., Dwivedi V.P., Maiga M., Maiga M., Van Kaer L., Bishai W.R., Das G. (2014). Small molecule-directed immunotherapy against recurrent infection by Mycobacterium tuberculosis. J. Biol. Chem..

[23] Bhattacharya D., Dwivedi V.P., Kumar S., Reddy M.C., Van Kaer L., Moodley P., Das G. (2014). Simultaneous inhibition of T helper 2 and T regulatory cell differentiation by small molecules enhances Bacillus Calmette-Guerin vaccine efficacy against tuberculosis. J. Biol. Chem..

[24] Pollard J.W. (2004). Tumour-educated macrophages promote tumour progression and metastasis. Nat. Rev. Cancer.

[25] Takayama H., Nishimura K., Tsujimura A., Nakai Y., Nakayama M., Aozasa K., Okuyama A., Nonomura N. (2009). Increased infiltration of tumor associated macrophages is associated with poor prognosis of bladder carcinoma in situ after intravesical bacillus Calmette-Guerin instillation. J. Urol..

[26] Ayari C., LaRue H., Hovington H., Decobert M., Harel F., Bergeron A., Têtu B., Lacombe L., Fradet Y. (2009). Bladder tumor infiltrating mature dendritic cells and macrophages as predictors of response to bacillus Calmette-Guérin immunotherapy. Eur. Urol..

[27] Hasita H., Komohara Y., Okabe H., Masuda T., Ohnishi K., Lei X.F., Beppu T., Baba H., Takeya M. (2010). Significance of alternatively activated macrophages in patients with intrahepatic cholangiocarcinoma. Cancer Sci..

[28] Hinz S., Pagerols-Raluy L., Oberg H.H., Ammerpohl O., Grüssel S., Sipos B., Grützmann R., Pilarsky C., Ungefroren H., Saeger H.D. (2007). FOXP3 expression in pancreatic carcinoma cells as a novel mechanism of immune evasion in cancer. Cancer Res..

[29] Miyake M., Gotoh D., Shimada K., Tatsumi Y., Nakai Y., Anai S., Torimoto K., Aoki K., Tanaka N., Konishi N. (2015). Exploration of risk factors predicting outcomes for primary T1 high-grade bladder cancer and validation of the Spanish Urological Club for Oncological Treatment scoring model: Long-term follow-up experience at a single institute. Int. J. Urol..

[30] Miyake M., Hori S., Morizawa Y., Tatsumi Y., Nakai Y., Anai S., Torimoto K., Aoki K., Tanaka N., Shimada K. (2016). CXCL1-Mediated Interaction of Cancer Cells with Tumor-Associated Macrophages and Cancer-Associated Fibroblasts Promotes Tumor Progression in Human Bladder Cancer. Neoplasia.

[31] Miyake M., Hori S., Morizawa Y., Tatsumi Y., Toritsuka M., Ohnishi S., Shimada K., Furuya H., Khadka V.S., Deng Y. (2017). Collagen type IV alpha 1 (COL4A1) and collagen type XIII alpha 1 (COL13A1) produced in cancer cells promote tumor budding at the invasion front in human urothelial carcinoma of the bladder. Oncotarget.

[32] Schwarz S., Butz M., Morsczeck C., Reichert T.E., Driemel O. (2008). Increased number of CD25+ FOXP3+ regulatory T cells in oral squamous cell carcinomas detected by chromogenic immunohistochemical double staining. J. Oral Pathol. Med..

